# EEG-Based Neurophysiological Assessment of Pharmacopoeial-Quality Essential Oils and Development of Stable Micro-Emulsion Systems for Aromatherapy Applications

**DOI:** 10.3390/ph19081291

**Published:** 2026-08-15

**Authors:** Melek Karaaslan, H. Kamuran İleri Özler, Burçin Ergene, Sevde Nur Biltekin Kaleli, Neslihan Üstündağ Okur, Simge Aykan, H. Gülçin Saltan İşcan

**Affiliations:** 1 Department of Pharmacognosy, Faculty of Pharmacy, Ankara University, Ankara 06560, Türkiye; ilerik@ankara.edu.tr (H.K.İ.Ö.); ergene@pharmacy.ankara.edu.tr (B.E.); gulcin.saltan@pharmacy.ankara.edu.tr (H.G.S.İ.); 2 Department of Pharmaceutical Microbiology, Faculty of Pharmacy, Medipol University, İstanbul 34810, Türkiye; snbiltekin@medipol.edu.tr; 3 Department of Pharmaceutical Technology, Faculty of Pharmacy, University of Health Sciences, İstanbul 34668, Türkiye; neslihanustundag.okur@sbu.edu.tr; 4 Department of Physiology, Faculty of Medicine, Ankara University, Ankara 06230, Türkiye; saykan@ankara.edu.tr; 5 GC Natura Herbal Products A.Ş., Ankara University Technopolis, Ankara 06830, Türkiye

**Keywords:** aromatherapy, cytotoxicity, Electroencephalography, essential oils, microemulsion formulations, neurophysiological responses

## Abstract

**Background/Objectives**: Despite the widespread use of essential oils in aromatherapy, objective evidence supporting their neurophysiological effects remains limited. This study evaluated the effects of selected pharmacopoeial-quality essential oils (lavender, rosemary, lemon, and peppermint as well as a blend of these oils) on electroencephalographic (EEG) activity following inhalation and developed and characterized microemulsion formulations guided by exploratory EEG response patterns, with preliminary in vitro cytotoxicity assessment. **Methods**: EEG recordings were obtained from 28 healthy volunteers following inhalation of the essential oils. Neurophysiological responses were evaluated using exploratory paired comparisons between each oil and its corresponding grape-seed oil control, together with repeated-measures ANOVA of normalized EEG responses. Selected oils were formulated as microemulsions (M1-M3), which were characterized by their physicochemical properties. Cytotoxicity was assessed in HaCaT human keratinocytes using the 3-(4,5-dimethylthiazol-2-yl)-2,5-diphenyltetrazolium bromide (MTT) assay. **Results**: Exploratory paired comparisons suggested localized EEG changes, particularly in the alpha-1 frequency band. However, these effects did not remain statistically significant after correction for multiple comparisons, and repeated-measures ANOVA of normalized EEG responses did not demonstrate significant overall differences among oil conditions, although the alpha-1 band exhibited the most distinct response pattern. Microemulsions exhibited nanoscale droplet sizes and favorable physicochemical stability. The IC_50_ values of M1 and M2 were greater than 2000 µg/mL, whereas M3 had an IC_50_ of approximately 1220 µg/mL. **Conclusions**: Pharmacopoeial-quality essential oils produced exploratory, region-specific EEG response patterns, with the alpha-1 band showing the most consistent response profile. The developed microemulsion formulations demonstrated suitable physicochemical stability and generally low cytotoxicity, supporting their further evaluation as aromatherapy product prototypes.

## 1. Introduction

Essential oils are secondary metabolites obtained from various aromatic plant species and consist of volatile, lipophilic, low-molecular-weight compounds. Extracted from plant tissues such as fruits, leaves, flowers, and roots, these essential oils comprise diverse chemical constituents, including hydrocarbons, aldehydes, ketones, alcohols, ethers, esters, phenols, oxides, and terpenes [1,2,3]. For millennia, essential oils have played a significant role in traditional medicine practices, spiritual ceremonies, cosmetics, aromatherapy, culinary practices, and food preservation [4].

Aromatherapy involves the therapeutic use of essential oils through various application methods, such as massage, inhalation, fomentation, baths, and foot baths [5,6]. Inhalation is particularly recognized for its effects on cognitive processes, including stress reduction, increased focus, sleep regulation, and improvement of dementia symptoms [5,6,7]. These effects are attributed to the ability of essential oil components to cross the lipid bilayer of cells, thereby enabling direct stimulation of the nervous system [8]. However, much of the knowledge regarding the biological activity of essential oils has been passed down through generations without scientific validation [9]. Therefore, it is essential to conduct studies examining their neurophysiological responses and their potential associations with cognitive functions using objective methodologies.

Electroencephalography (EEG) has emerged as a valuable tool for evaluating neurophysiological responses to essential oils. Numerous studies using EEG analyses have investigated neurophysiological responses to essential oils and reported changes in EEG activity that have been associated with alterations in mood, physiology, and behavior through interactions with the central nervous system [1,7,10,11,12]. EEG records the brain’s electrical activity via scalp electrodes and serves as a quantitative indicator of brain states. The traditional EEG frequency bands are commonly investigated in relation to different neurophysiological states and may be associated with cognitive or emotional processes [1].

In a pioneering study, EEG measurements obtained from 20 healthy volunteers during inhalation of lavender essential oil revealed increased theta (4–8 Hz) and alpha (8–13 Hz) power, particularly in the bilateral temporal and central areas [13]. Another study, conducted with 12 volunteers, showed that inhalation of peppermint essential oil significantly reduced alpha wave activity across almost all lobes [6]. Additionally, two studies on rosemary essential oil reported a reduction in alpha wave activity [13,14].

Several essential oils have been extensively used for their potential neurological and therapeutic properties. Lavender (*Lavandula angustifolia* Mill.), a member of the Lamiaceae family, is widely cultivated for its essential oil, which is used in aromatherapy to manage conditions such as aggression, insomnia, spasms, pain, headache, depression, and anxiety [15,16,17]. Peppermint (*Mentha* × *piperita* L.), also from the Lamiaceae family, is commonly used for oral and dental care, headache relief, and respiratory conditions. Its essential oil exhibits anti-inflammatory, antioxidant, antimicrobial, and cognitive-enhancing properties [18,19]. Rosemary (*Rosmarinus officinalis* L.), another Lamiaceae species, is used in aromatherapy for its anti-inflammatory and antidepressant effects, as well as its role in improving lymphatic flow [15,20].

The essential oil obtained from lemon (*Citrus limon* (L.) Osbeck), a member of the Rutaceae family, has been reported to enhance memory, concentration, cognitive performance, attention, and mood [21,22]. However, essential oils are chemically sensitive to environmental factors such as temperature, air, humidity, and light. Thus, in addition to quality control studies, formulation development strategies are crucial to improve product stability [23].

Innovative nanocarrier systems, including liposomes, solid lipid nanoparticles, niosomes, dendrimers, nanoemulsions, and nanospheres, have been employed to enhance the dermal penetration and controlled delivery of essential oils in topical formulations. These nanocarriers enhance the stability and bioavailability of active components, making them suitable for pharmaceutical and cosmetic applications [24]. Microemulsions are thermodynamically stable, nanostructured colloidal dispersions composed of water and oil phases. These systems, typically featuring droplet diameters between 10 and 100 nm, are stabilized through the incorporation of surfactants and co-surfactants and may adopt various structural forms such as oil-in-water, water-in-oil, or bicontinuous configurations [25,26]. Although microemulsions require high concentrations of surfactants, their benefits, such as ease of manufacturing, high ingredient loading capacity, and improved stability, make them advantageous for drug delivery [27].

The increasing use of essential oils highlights the importance of ensuring their quality, safety, and formulation stability for aromatherapy applications [28]. Although previous studies have investigated the neurological effects of essential oils, most have focused solely on descriptive outcomes without integrating formulation development or establishing an objective strategy for selecting oils for subsequent formulation. Therefore, a critical gap remains in linking EEG-based neurophysiological assessment with industrially applicable formulation design. Within this framework, neurophysiological responses to the inhaled essential oils were explored using EEG, whereas the physicochemical properties and preliminary in vitro cytotoxicity of the subsequently developed microemulsion formulations were evaluated separately. Since the properties of aromatherapy products are directly related to essential oil composition, the essential oils used in this study had previously undergone pharmacopoeial quality testing and GC–MS characterization, confirming their compliance with pharmaceutical-grade standards [29]. EEG analyses were conducted to assess neurophysiological responses elicited by inhalation of these essential oils. The selected essential oils were subsequently incorporated into microemulsion formulations. For preliminary in vitro safety screening, the developed microemulsion formulations were evaluated using the MTT assay. This study presents an integrated approach in which EEG was used as an exploratory tool to characterize neurophysiological responses prior to formulation development. By combining EEG assessment, microemulsion development, and in vitro safety evaluation, the proposed framework supports the development of stable, low-toxicity aromatherapy formulations based on pharmacopoeial-quality essential oils. The exploratory EEG observations informed the selection of oils for formulation development; however, the developed formulations themselves were not evaluated in the EEG experiment.

## 2. Results and Discussion

### 2.1. EEG Data

#### 2.1.1. Theta Band

Analysis of the experimental data revealed that among all tested oils, only lemon essential oil produced a nominally significant alteration in relation to its control condition. Specifically, when compared with the grape-seed oil control condition, lemon oil elicited an increase in EEG activity in the left frontal region (Mean difference: 0.0190; *p* = 0.031) and left temporal region (Mean difference: 0.0192; *p* = 0.045) in the theta band (Table 1).

A 5 × 2 × 2 repeated-measures ANOVA was conducted on log-transformed theta power ratios, with oil type, cortical region, and hemisphere as within-subject factors. Mauchly’s test showed that sphericity was not violated for oil type, *W* = 0.610, χ^2^(9) = 12.57, *p* = 0.184. The main effects of oil type, *F*(4, 108) = 1.73, *p* = 0.149, ηp^2^ = 0.060, cortical region, *F*(1, 27) = 0.88, *p* = 0.356, ηp^2^ = 0.032, and hemisphere, *F*(1, 27) = 2.09, *p* = 0.160, ηp^2^ = 0.072, were not significant.

None of the interaction effects reached statistical significance, including oil × region, *F*(4, 108) = 1.02, *p* = 0.400, oil × hemisphere, *F*(4, 108) = 0.61, *p* = 0.660, and oil × region × hemisphere, *F*(4, 108) = 0.94, *p* = 0.444. Although lemon oil showed the largest positive mean theta change, *M* = 0.013, *SE* = 0.005, 95% CI [0.002, 0.023], Bonferroni-adjusted comparisons revealed no significant differences between oil conditions. Overall, theta power was not systematically modulated by oil type, cortical region, hemisphere, or their interactions.

Taken together, the repeated-measures ANOVA and paired-samples *t*-tests indicate that theta power was not broadly or systematically affected by oil type, cortical region, or hemisphere. Exploratory paired comparisons showed small increases following lemon oil in the left frontal and left temporal regions; however, these effects did not remain significant after correction for multiple comparisons. Thus, the theta findings suggest a limited, region-specific pattern associated with lemon oil rather than a robust overall modulation of theta activity.

#### 2.1.2. Alpha-1 Band

A notable reduction in alpha-1 band activity was observed in the left frontal region for lavender, mixed and peppermint oils (Table 2).

The lavender oil resulted in a nominal decrease in alpha-1 activity in the left frontal region (Table 2, Mean difference: −0.0235; *p* = 0.017) and the right temporal region (Table 2, Mean difference: −0.0326; *p* = 0.020), relative to the control condition (grape-seed oil).

The mixture resulted in a nominal decrease in alpha-1 activity at the right frontal region (Table 2, Mean difference: −0.0206; *p* = 0.004) and left frontal region (Table 2, Mean difference: −0.0188; *p* = 0.048) relative to control condition (grape-seed oil).

Peppermint essential oil showed a nominal decrease in alpha-1 activity at the right frontal region (Table 2, Mean difference: −0.0214; *p* = 0.015) in relation to the grape-seed oil control group.

A 5 × 2 × 2 repeated-measures ANOVA was conducted on the log-transformed alpha-1 power ratios (target oil/target oil control), with oil type (lavender, rosemary, mixed oil, lemon, and peppermint), cortical region (frontal and temporal), and hemisphere (right and left) entered as within-subject factors for comparison between different oils. Mauchly’s test indicated that the assumption of sphericity was violated for the main effect of oil, *W* = 0.454, χ^2^(9) = 20.10, *p* = 0.018; therefore, Greenhouse–Geisser-corrected results were reported. The main effect of oil type on alpha-1 power did not reach statistical significance, *F*(2.99, 80.82) = 2.67, *p* = 0.053, ηp^2^ = 0.090. The main effects of cortical region, *F*(1, 27) = 0.77, *p* = 0.388, ηp^2^ = 0.028, and hemisphere, *F*(1, 27) = 0.91, *p* = 0.349, ηp^2^ = 0.033, were also not significant. Furthermore, none of the interaction effects reached statistical significance: oil × region, *F*(2.77, 74.71) = 1.49, *p* = 0.227, ηp^2^ = 0.052; oil × hemisphere, *F*(2.26, 61.13) = 0.37, *p* = 0.715, ηp^2^ = 0.014; region × hemisphere, *F*(1, 27) = 0.35, *p* = 0.557, ηp^2^ = 0.013; or oil × region × hemisphere, *F*(2.33, 63.03) = 0.67, *p* = 0.537, ηp^2^ = 0.024. Bonferroni-adjusted pairwise comparisons likewise revealed no significant differences between the individual oil conditions.

In summary, alpha-1 power showed no robust oil-specific, regional, or hemispheric modulation in the omnibus analysis. Exploratory paired comparisons indicated localized reductions in alpha-1 power, particularly following mixed-oil exposure in the right frontal region; however, most of these effects did not remain significant after correction for multiple comparisons.

#### 2.1.3. Alpha-2 Band

Within the alpha-2 frequency range, lavender essential oil was found to nominally reduce neural activity in the right temporal area (Table 3, Mean difference: −0.0377; *p* = 0.027) compared with the control grape-seed group.

A 5 × 2 × 2 repeated-measures ANOVA was conducted on the log-transformed alpha-2 power ratios, with oil type (lavender, rosemary, mixed oil, lemon, and peppermint), cortical region (frontal and temporal), and hemisphere (right and left) entered as within-subject factors. Mauchly’s test indicated that the assumption of sphericity was not violated for the main effect of oil, *W* = 0.512, χ^2^(9) = 16.33, *p* = 0.061; therefore, the sphericity-assumed results were reported. The main effect of oil type on alpha-2 power was not statistically significant, *F*(4, 104) = 0.82, *p* = 0.518, ηp^2^ = 0.030. The main effect of cortical region also did not reach statistical significance, although a nonsignificant tendency toward regional differences was observed, *F*(1, 26) = 3.05, *p* = 0.092, ηp^2^ = 0.105. The main effect of hemisphere was not significant, *F*(1, 26) = 0.18, *p* = 0.679, ηp^2^ = 0.007.

None of the interaction effects reached statistical significance: oil × region, *F*(4, 104) = 1.56, *p* = 0.192, ηp^2^ = 0.056; oil × hemisphere, *F*(4, 104) = 1.36, *p* = 0.253, ηp^2^ = 0.050; region × hemisphere, *F*(1, 26) = 0.42, *p* = 0.522, ηp^2^ = 0.016; or oil × region × hemisphere, *F*(4, 104) = 1.56, *p* = 0.190, ηp^2^ = 0.057. Bonferroni-adjusted pairwise comparisons likewise revealed no significant differences between the individual oil conditions. Overall, these findings indicate that alpha-2 power was not systematically modulated by oil type, cortical region, or hemisphere.

Considered together, no significant and systematic changes in alpha-2 band power were observed as a function of oil type, cortical region, or hemisphere. Exploratory paired comparisons indicated that lavender oil was associated with a small reduction in alpha-2 power in the right temporal region relative to the grape-seed oil control condition; however, this localized finding did not remain significant after correction for multiple comparisons. Therefore, the alpha-2 findings suggest a limited and region-specific pattern that requires further confirmation rather than a reliable modulation associated with essential oil exposure.

#### 2.1.4. Exploratory Sex-Based Differences Analysis

Exploratory sex-based analyses in the alpha-1 band did not reveal a statistically significant difference between female and male participants. Although lavender essential oil showed numerically greater alpha-1 changes in female participants than in males, these differences did not reach statistical significance. Therefore, no sex-specific effect can be inferred from the present data.

### 2.2. Microemulsion Formulation and Characterization

The formulation-selection process was conducted within a technology development project and was informed by the initial exploratory pairwise EEG observations, the intended product concepts, and the reported aromatherapy uses of the oils. Lavender was selected for the pillow-spray and topical-patch prototypes because the initial analyses showed localized alpha-1 and alpha-2 changes and because of its common use in relaxation- and sleep-oriented aromatherapy applications. The rosemary–lemon–peppermint mixture was selected as a representative multi-oil prototype intended for alertness- and cognition-oriented applications. This selection was also informed by the relatively broader localized alpha-1 response pattern initially observed for the mixture. Lemon, peppermint, and rosemary were not formulated separately because the project was limited to representative single-oil and multi-oil proof-of-concept prototypes. Since attention and cognitive performance were not directly assessed and the multiplicity-adjusted EEG analyses did not confirm significant oil-specific effects, these EEG observations were treated as exploratory findings that informed formulation selection but did not constitute functional validation of the developed products. The developed formulations themselves were not administered during EEG recording; therefore, comparable exposure conditions and neurophysiological responses have not yet been demonstrated for either the pillow-spray or topical-patch prototypes.

**Figure 1:**
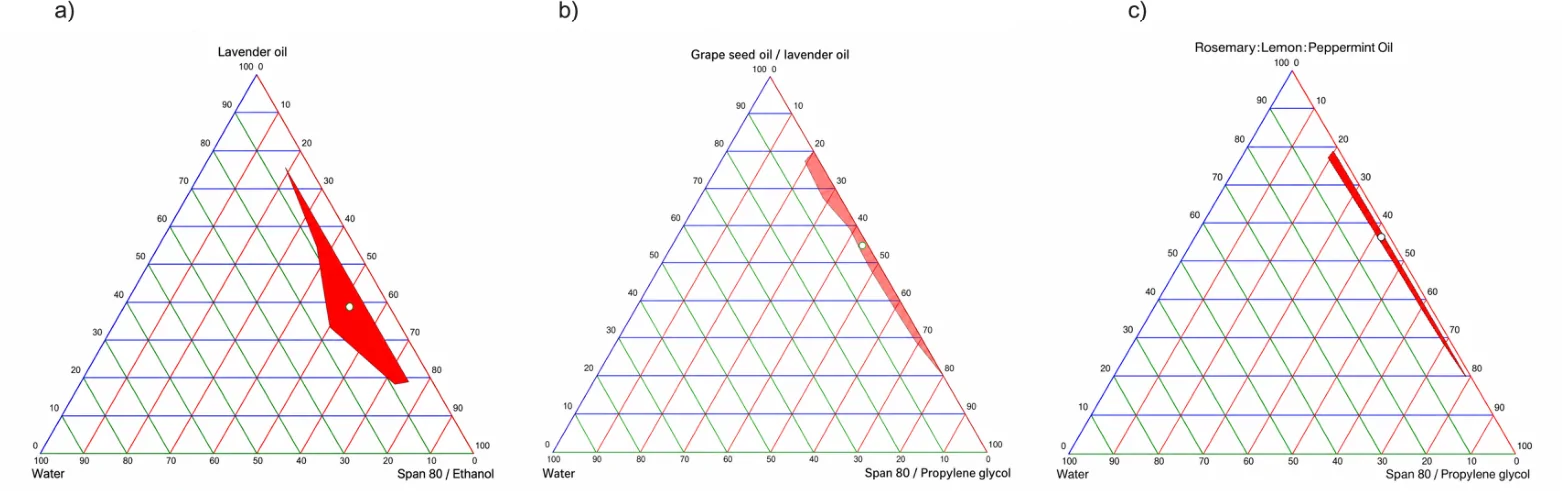
The pseudo-ternary phase diagrams of microemulsions. (**a**) The pseudo-ternary phase diagram of the **M1** microemulsion composed of lavender oil, Span 80/ethanol and water (w/o). (**b**) The pseudo-ternary phase diagram of the **M2** microemulsion composed of grape-seed oil/lavender oil, Span 80/propylene glycol and water (w/o). (**c**) The pseudo-ternary phase diagram of the **M3** microemulsion composed of rosemary/lemon/peppermint oil mixture (1:1:1 ratio), Span 80/propylene glycol and water (w/o). The colored grid lines indicate the percentage scales of the three components in the pseudo-ternary phase diagram. The red-shaded areas indicate the single-phase microemulsion regions, and the white dots represent the selected optimum formulations.

Following the selection of the representative single-oil and multi-oil systems, preformulation studies were conducted to determine the optimal microemulsion compositions. To determine the optimal formulation, the maximum amount of water that could be added without causing visible phase separation during titration was identified. Before the preparation stage, it is necessary to plan the components required for the desired microemulsion type and the properties expected from the formulation. After this stage, it is necessary to examine the different phase behaviors using the phase diagram, which helps the researchers reach their goal [30]. The triangular phase diagram serves as a practical reference for identifying suitable microemulsion composition ranges [31]. In the triangular phase diagram, each corner of the triangle represents one component at 100% by volume. When moving away from a corner, the volumetric proportion of the component represented at that corner decreases, while the proportion of one or both other two components increase. According to Gibbs’ phase rule, each point in the triangle represents a possible composition of the mixture, which can consist of one, two or three phases [32]. A systematic, stepwise titration protocol was employed to effectuate the elaboration of the microemulsion formulations. The formulation optimization process was performed using pseudo-ternary phase diagrams, and the optimal compositions were selected from the centroid of the largest microemulsion region. For M1, surfactant/co-surfactant ratios of 1:1, 1:2, 1:3, 1:4, and 1:5 were investigated, whereas ratios of 1:1, 2:1, 3:1, 4:1, and 5:1 were evaluated for M2 and M3. The final formulations were selected according to the pseudo-ternary phase diagram analysis and the largest microemulsion region. The pseudo-ternary phase diagrams and the selected optimum formulation points are shown in Figure 1.

The compositions of the selected M1, M2, and M3 formulations are presented in Table 4.

Freshly prepared microemulsion prototypes M1, M2, and M3 were subjected to comprehensive physicochemical characterization, whereby pH, specific conductivity, refractive index, apparent viscosity, polydispersity index, zeta-potential, and mean droplet diameter were systematically quantified. Visual appraisal for turbidity, opalescence, and incipient phase boundaries—followed by centrifugal stress testing—was performed for each formulation. To interrogate the anticipated thermodynamic robustness of the blank microemulsion matrix, samples were subjected to ultracentrifugation at 13,000 rpm (≈16,000 g, fixed-angle rotor) for 30 min at 25 ± 2 °C as an integral component of the characterization protocol. Post-centrifugation inspection disclosed no evidence of creaming, sedimentation, or phase delamination in any of the formulations, thereby corroborating their isotropic stability under high-shear conditions. Following ultracentrifugation at 20,000 rpm, the microemulsion formulated by Kharwade et al. reported that the emulsion showed no evidence of phase separation, leading the authors to designate the system as physically stable [33]. Because pH critically influences both biocompatibility and therapeutic performance, the pH of the present prototypes—M1, M2, and M3—was determined, yielding values between 6.7560 ± 0.0500 and 7.0400 ± 0.0054 (Table 5). Diagnostic insight into formulation architecture can be garnered from the electrical conductivity recorded by conductometric analysis. A negligible conductance indicates an oil-continuous microemulsion, whereas a comparatively high conductivity indicates water as the external (continuous) phase [34]. Conductometric profiling of the present microemulsion prototypes revealed electrical conductivities ranging from 0 to 0.0365 ± 0.00007 μS, a range that signifies an oil-continuous external phase (Table 5).

Refractometric analysis established refractive indices ranging from 1.4182 ± 0.0002 and 1.46588 ± 0.0002 for the formulations (Table 5); these values subsequently served as input parameters for calculating mean droplet diameter. Viscometric measurements of the same prototypes (Table 5) revealed uniformly low apparent viscosities, spanning 4.520 ± 0.042 to 40.100 ± 7.449 cP.

The practical utility of a microemulsion is largely influenced by the magnitude of its dispersed-phase droplets. Accordingly, Table 5 presents the mean droplet diameters alongside their corresponding polydispersity indices for each formulation. Polydispersity index values very close and below 0.5 reflect a uniform distribution of droplet sizes within the formulation, indicating good homogeneity [35]. Hydrodynamic diameters for the dispersed droplets ranged from 1.106 ± 0.015 nm to 92.19 ± 11.053 nm. Moreover, polydispersity indices remained below 0.5 for every formulation, indicating a homogeneous particle-size distribution and supporting the pharmaceutical suitability of the nanometric dimensions. Droplet sizes in microemulsions vary; in particular, the M1 formulation was found to have a size of 1.106 ± 0.015 nm and a PDI value of 0.209 ± 0.017. Microemulsions containing droplets of similar sizes have been reported in similar studies in the literature [36,37,38]. The selection and ratios of the surfactants in the microemulsion’s composition play a critical role. In this study, the small droplet size of M1, in particular, is attributed to the presence of ethanol. Ethanol’s ability to increase interfacial fluidity and reduce droplet size has brought the droplet size down to approximately 1 nm while also ensuring homogeneity with suitable PDI values [39]. Particle surface-charge characteristics were evaluated by ζ-potential analysis—an established metric for appraising microemulsion stability—with recorded values ranging from –0.057 ± 0.0021 mV to –0.414 ± 0.515 mV (Table 5). These results imply that nonionic microemulsion systems are stabilized predominantly through steric rather than electrostatic mechanisms.

After 3 months of storage, all formulations retained a suitable appearance under the three tested conditions; the corresponding physicochemical results are summarized in Table 6.

### 2.3. Cytotoxicity Analyses

Viability tests were carried out on HaCaT human skin keratinocyte cells to evaluate the cytotoxic effects of the formulations, in three independent experiments, each performed in triplicate. The control group in the experiments was designated as the group receiving no agent application and was considered 100% viable. One-way ANOVA was applied to the obtained data to analyze the consistency between repetitions within the experimental groups. To assess statistical significance in relation to the control group, both Dunnett’s and Tukey’s post hoc tests were applied. Based on the literature reviews, decisions regarding the concentrations and exposure times of the agents were made. The half-maximal inhibitory concentration values (IC_50_) were calculated for the applied formulations. The IC_50_ values of the agents were calculated using the sigmoidal dose–response curve in GraphPad Prism^®^ 7.0 software.

#### Results of Cytotoxic Analysis for M1 (Pillow Spray), M2 (Lavender Topical Patch), M3 (Rosemary:Lemon:Peppermint Topical Patch)

**Figure 2:**
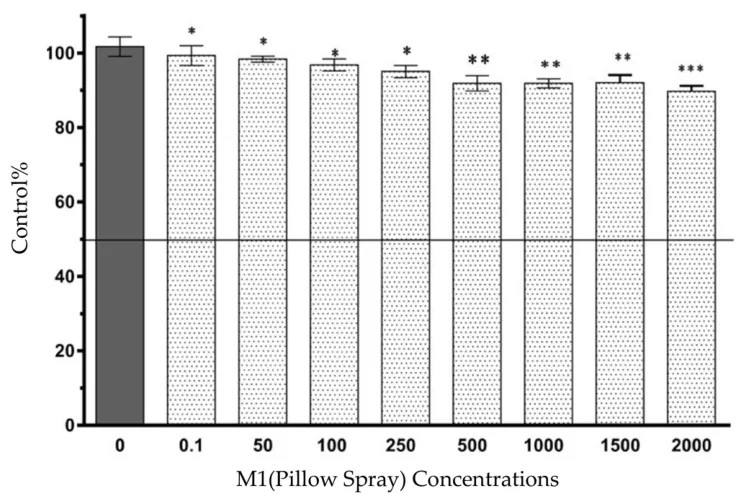
The effect of 24-h exposure to M1 (Pillow Spray) at various concentrations (μg/mL) on the cell viability of HaCaT human skin keratinocytes. Data are expressed as percentage of the untreated control (0 μg/mL) and represent the mean ± standard deviation (SD) from three independent experiments performed in triplicate (*n* = 9). Statistical significance was determined using one-way ANOVA followed by Dunnett’s post hoc test (* p < 0.05, ** p < 0.01, *** p < 0.001, vertical bars indicate standard deviation values).

**Figure 3:**
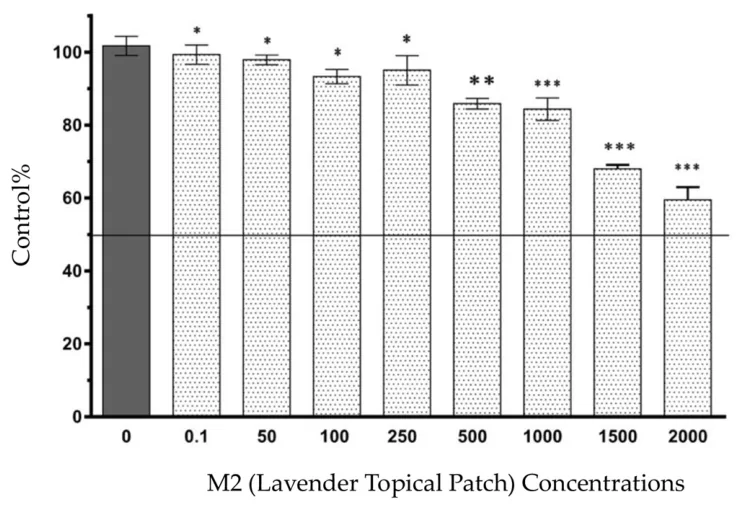
The effect of 24-h exposure to M2 (Lavender Topical Patch) at various concentrations (μg/mL) on the cell viability of HaCaT human skin keratinocytes. Data are expressed as percentage of the untreated control (0 μg/mL) and represent the mean ± standard deviation (SD) from three independent experiments performed in triplicate (*n* = 9). Statistical significance was determined using one-way ANOVA followed by Dunnett’s post hoc test (* p < 0.05, ** p < 0.01, *** p < 0.001, vertical bars indicate standard deviation values).

**Figure 4:**
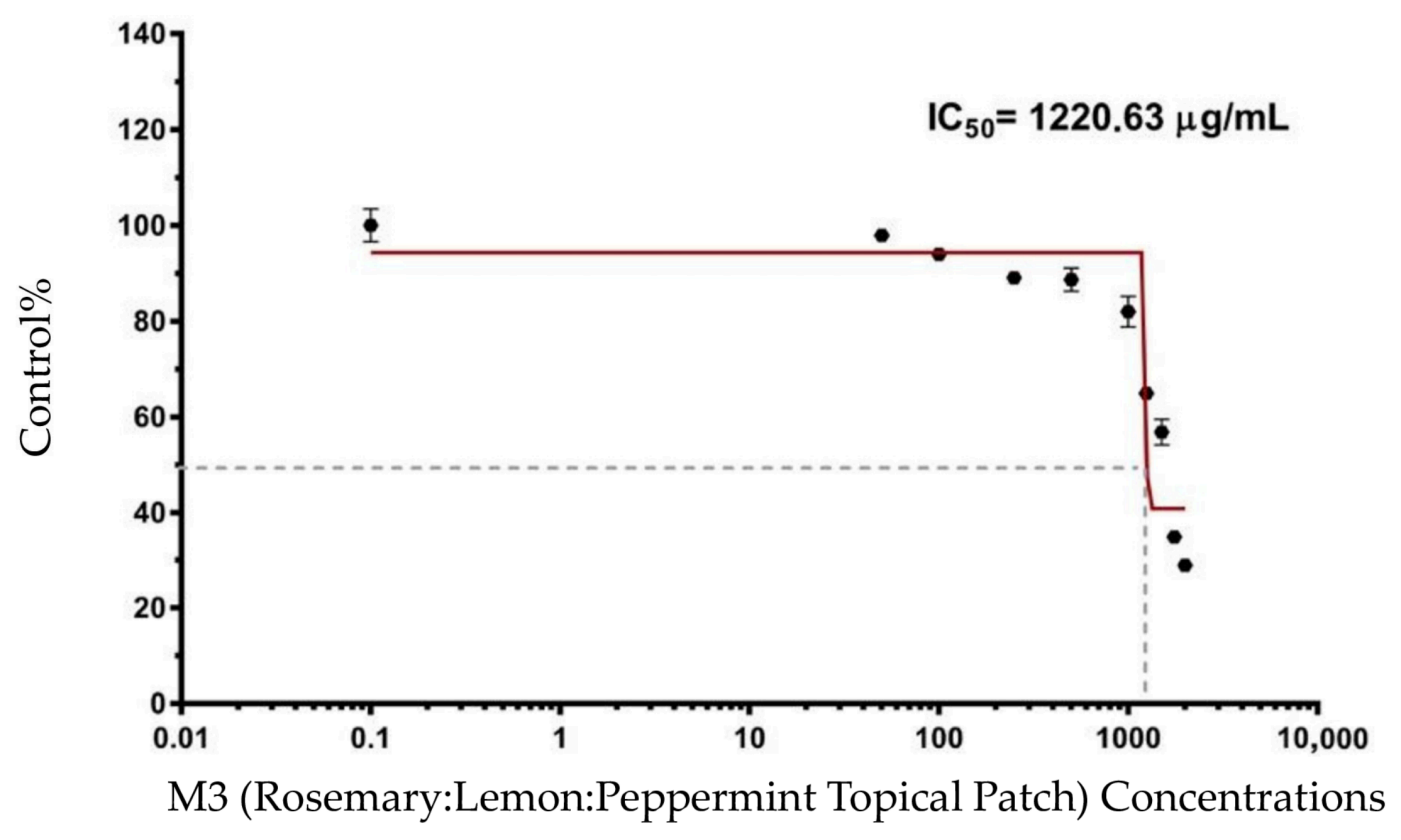
Non-linear regression dose–response curve for the determination of ${\mathrm{IC}}_{50}$ of M3 (Rosemary:Lemon:Peppermint Topical Patch) on HaCaT human skin keratinocyte following 24-h exposure (*r*^2^ = 0.9703). Data points represent the mean ± standard deviation (SD) of three independent experiments performed in triplicate (*n* = 9), expressed as a percentage of viability relative to the untreated control. Concentrations (μg/mL) are plotted on a logarithmic scale.

To assess the cytotoxic impact of the three formulations on HaCaT human keratinocyte cells, the cultures were treated for 24 h with media containing test substances at graded concentrations of 0.1, 50, 100, 250, 500, 1000, 1500, and 2000 µg/mL. For the control group with no test substance applied, the culture medium was renewed. After 24 h of incubation period, data were obtained through the MTT test. The results for the 24-h effects of M1 (Pillow Spray), M2 (Lavender Topical Patch), and M3 (Rosemary:Lemon:Peppermint Topical Patch) on cells are presented in Figure 2, Figure 3 and Figure 4. The concentrations of M1 and M2 that reduced cell viability by 50% within 24 h could not be calculated within the tested concentration range and are reported as IC_50_ > 2000 µg/mL (IC_50_ not reached up to 2000 µg/mL). The IC_50_ value of the positive control cisplatin against HaCaT cells was determined as 14.36 µg/mL.

According to the analysis conducted following a 24-h application, the concentration of M3 that reduced cell viability by 50% (IC_50_) was determined to be 1220.63 µg/mL (95% Confidence Interval: 1145.20 to 1301.12 µg/mL; calculated using a non-linear regression model with top/bottom fitting constraints set to 100% and 0% respectively, R^2^ = 0.9703).

It must be emphasized that while the HaCaT MTT assay provides valuable and essential preliminary screening data regarding cellular metabolic activity and potential cytotoxicity, it is not a validated skin-irritation assay and cannot conclusively demonstrate comprehensive skin compatibility or clinical safety. Furthermore, because HaCaT keratinocytes represent epidermal tissue, these in vitro results do not provide meaningful safety data regarding the inhalation toxicity or airway irritation potential of the M1 pillow spray formulation. Nevertheless, these findings successfully establish a foundational baseline for the comparative safety of the formulations. To build upon this proof-of-concept, further specific in vitro inhalation models or in vivo safety evaluations are required to fully delineate the respiratory safety profile of the developed spray.

A limitation of the present cytotoxicity assessment is that blank microemulsions, matched free essential oils, surfactant/co-surfactant systems, and vehicle controls were not included; therefore, the individual contributions of the formulation components to the observed cell-viability responses could not be distinguished.

Electrophysiological studies have investigated whether specific scents modulate spontaneous brain activity and cognitive processes. For example, reductions in alpha and beta wave patterns, coupled with elevations in delta and theta frequencies, are often linked to neurological dysfunction and cognitive impairment. Numerous studies have employed EEG to investigate how olfactory stimuli affect neural dynamics and mental performance. Previous studies have examined associations between odor exposure, EEG activity, mood-related variables, and cognitive outcomes [40,41]. In other studies where essential oils were administered via inhalation and their effects were examined with EEG, significant changes at alpha, beta, and theta band levels were generally observed in the frontal, temporal, and parietal regions [13,40,42,43,44]. Overall, the present findings contribute to the existing literature on EEG responses to essential oil inhalation by identifying small, exploratory EEG changes under the present experimental conditions; however, these findings should not be interpreted as confirmatory evidence of oil-specific neurophysiological effects.

To comprehensively evaluate the EEG responses, two complementary analytical approaches were applied. The five test conditions consisted of four individual essential oils (lavender, rosemary, lemon, and peppermint) and one mixed essential oil formulation. First, each test condition was compared with its matched grape-seed oil control to identify condition-specific EEG responses. Subsequently, EEG responses normalized to the corresponding grape-seed oil control condition were compared across the five test conditions to determine whether different test conditions produced distinct neurophysiological response patterns.

In the pairwise comparisons, a small overall reduction in alpha-1 power was observed across the test conditions relative to their matched grape-seed oil controls. Exploratory comparisons indicated that this reduction was more pronounced following lavender and mixed essential oil inhalation. Several nominally significant regional findings were observed; however, none remained statistically significant after Bonferroni correction for 20 comparisons. Therefore, the findings obtained from the pairwise comparisons should be interpreted cautiously.

The repeated-measures ANOVA of the normalized alpha-1 responses revealed no statistically significant main effects of test condition, cortical region, or hemisphere and no significant interaction effects. Nevertheless, the main effect of test condition remained borderline after Greenhouse–Geisser correction (*p* = 0.053). Among the frequency bands examined, alpha-1 showed the clearest overall tendency; however, the findings should be regarded as exploratory and hypothesis-generating rather than confirmatory.

Lavender essential oil has long been investigated in relation to relaxation, stress relief, and sleep quality [45]. Previous EEG studies examining lavender inhalation have also reported changes in alpha-band activity [13,41,43,45,46]. Alpha activity has often been associated with relaxed wakefulness and internally directed attention, whereas beta activity has generally been associated with active cognitive engagement and externally oriented attention. Nevertheless, the functional interpretation of EEG frequency bands depends strongly on the experimental context [47].

Previous studies do not provide a clear consensus regarding whether alpha-band activity increases or decreases following lavender essential oil inhalation. Therefore, the reduction in alpha-1 activity observed in the present pairwise analyses cannot alone be interpreted as evidence of a sedative or relaxation-related effect. Koulivand et al. reported significant changes in left frontal EEG activation following lavender essential oil inhalation in 39 adults [45]. The present findings indicate a tendency toward alpha-1 modulation following lavender inhalation, but this effect was not confirmed after correction for multiple comparisons. Further studies are therefore needed to clarify the direction and functional significance of alpha-band responses to lavender inhalation.

When the results obtained for the mixture are considered in the light of the literature, studies investigating rosemary with EEG are more prominent. Sayorwan et al. evaluated the effects of rosemary essential oil using EEG and reported a decrease in alpha-band power [44]. Studies on rosemary oil generally focus on the beta-wave activity [48]. Additionally, there are a few studies on the changes occurring in the alpha-band for lemon and peppermint essential oils, which are also components of the mixture.

Park et al. investigated how peppermint essential oil influences EEG activity, specifically focusing on alpha and beta bands recorded at Fp1, Fp2, F3, F4, P4, and P3 electrode sites. Their results demonstrated a reduction in alpha-band power following peppermint oil exposure [6].

Lemon essential oil is recognized in the literature for its use as a relaxant in anxiety and sleep disorders, as well as for its role in enhancing cognitive processes. Notably, similar to lavender essential oil, it stands out for its anxiolytic effects and its application as a calming agent [49,50,51].

Ueda et al. investigated the neural effects of lemon essential oil and reported increased delta and theta activity in prefrontal regions—such as the anterior cingulate, orbitofrontal cortex, superior temporal gyrus, parahippocampal area, and insula—within the first 60 s of inhalation. Additionally, alpha-band activation emerged in the anterior cingulate immediately following exposure, with more widespread prefrontal engagement observed between 90 and 120 s post-inhalation [7].

In the present study, exploratory pairwise comparisons showed nominal reductions in alpha-1 power following peppermint inhalation in the right frontal region and following mixed essential oil inhalation in the right and left frontal regions. However, none of these regional findings remained statistically significant after correction for multiple comparisons. Therefore, the present results do not provide evidence that the mixed-oil condition produced a broader regional response or was more effective than the individual essential oils, and no inference regarding synergistic effects can be made.

The alpha-2 analyses did not reveal statistically significant main effects of test condition, cortical region, or hemisphere, and none of the interaction effects reached statistical significance. An exploratory pairwise comparison suggested a small reduction in alpha-2 power in the right temporal region following lavender inhalation relative to its matched grape-seed oil control; however, this localized finding did not remain statistically significant after correction for multiple comparisons. Therefore, the present findings do not establish a reliable oil-specific effect on alpha-2 power.

In the present study, potential sex-related differences in EEG responses were also considered. Prior research indicates that sex-based variations may influence EEG outcomes, as male and female brains may exhibit different patterns of hemispheric lateralization during cognitive processing [41]. However, exploratory sex-stratified analyses did not reveal statistically significant sex-related differences in EEG responses. Therefore, no sex-specific conclusion can be drawn from the present dataset. Consistent with this finding, a previous study comparing lavender oil with sweet almond oil also reported no statistically significant sex-based differences in mean values [13]. Future studies with larger and adequately powered sex-stratified samples are needed to clarify whether sex influences EEG responses to essential oil inhalation.

The present study has several limitations that should be considered when interpreting the findings. Although the order of the five essential-oil conditions was randomized across participants, the sequence within each control–odor pair was fixed, with the grape-seed oil control always preceding the corresponding essential oil. Consequently, potential order-related effects, including changes in vigilance, fatigue, olfactory adaptation, habituation, or cumulative exposure, cannot be ruled out. Future studies employing fully counterbalanced crossover designs with randomized control and odor presentation sequences would help minimize these potential confounding effects.

No objective cognitive or behavioral assessments, such as memory, attention, or performance tasks, were conducted alongside EEG recordings. Therefore, the observed nominal patterns in theta and alpha activity should be interpreted as exploratory neurophysiological responses rather than direct evidence of specific cognitive or emotional outcomes. Complete participant blinding was not feasible because of the characteristic odors of the essential oils. Although grape-seed oil was used as a vehicle control, it did not constitute an odor-matched control for the strong-smelling essential oils. Similar carrier-oil controls have been employed in previous inhalation EEG studies [13,44]; however, such controls do not fully account for nonspecific olfactory stimulation. Furthermore, odor recognition, perceived intensity, pleasantness, familiarity, and expectancy were not assessed. Accordingly, expectancy effects and the potential influence of these factors on the observed EEG responses cannot be entirely excluded.

The relatively small sample size limited the statistical power of the study, particularly for sex-stratified analyses; therefore, sex-related effects should be evaluated in future studies with larger and more balanced cohorts. Consequently, the generalizability of the findings to other populations and clinical settings remains limited and the results should be interpreted with appropriate caution. In addition, multiple EEG comparisons were performed across frequency bands, regions, and oil conditions; therefore, the findings should be interpreted as exploratory EEG-based screening results rather than confirmatory neurophysiological evidence.

Finally, EEG responses were evaluated following the experimental inhalation of the essential oils, whereas the developed formulations themselves were not evaluated in the EEG experiment. The topical-patch prototypes involve a different route of exposure, while the pillow-spray prototype may produce inhalation conditions that differ from those used in the experimental procedure. Therefore, comparable exposure conditions and neurophysiological responses have not yet been demonstrated for the developed formulations. Direct formulation-specific studies in human participants are required before any functional effects of the final products can be inferred.

The novelty of this study lies in its hypothesis-driven integration of EEG-based neurophysiological assessment with formulation development. Unlike previous studies that have mainly reported descriptive EEG findings on essential oils, the present study used EEG not only to assess neurophysiological responses but also to provide an objective approach for selecting essential oils for subsequent formulation development.

The findings demonstrate that essential oils selected with reference to exploratory EEG observations can subsequently be incorporated into physicochemically stable microemulsion systems with preliminary profiles of low cytotoxicity. However, the EEG assessment and formulation evaluation represent sequential but distinct stages of the study. In this respect, the study provides an integrated framework linking exploratory neurophysiological assessment with formulation development, thereby supporting the development of pharmacopoeial-quality essential oil formulations for aromatherapy applications.

## 3. Materials and Methods

### 3.1. Participants

Thirty healthy volunteers, including an equal number of males and females, participated in the study. The study sample consisted of individuals aged 18 to 35 years who had a body mass index within the normal range of 20–25 kg/m^2^. All participants were confirmed as right-handed based on the validated Turkish adaptation of the Hand Preference Questionnaire [52,53]. Only individuals with intact olfactory function were enrolled in the study. A diagnosis of asthma, migraine, hypertension, or cardiovascular disorders was considered an exclusion criterion. Olfactory sensitivity was assessed using an adaptation test based on tachyphylaxis principles. Participants with a history of substance abuse or use of tobacco products were not eligible for inclusion. Additionally, women who were pregnant or breastfeeding were excluded. The follicular phase was determined based on the self-reported first day of the last menstrual period, and EEG recordings were scheduled according to calendar-based menstrual cycle calculations. Only women in the follicular phase were included in the study.

### 3.2. Study Design

The participants underwent an olfactory adaptation test the day before and participated in the study the following day. During their initial visit to the Ankara University Brain Research Center, the participants who agreed to participate in the study signed an informed consent form. Afterward, they completed the sociodemographic form and the Handedness Scale. For psychological assessment, participants completed the Beck Anxiety Inventory [54,55], Beck Depression Inventory [56], and the Turkish version of the Adult ADD/ADHD DSM-IV-Based Diagnostic Screening and Rating Scale [57,58]. After completion of the questionnaires, EEG data were subsequently recorded.

### 3.3. Measurement Tools

#### 3.3.1. Olfactory Adaptation Test

To determine the duration of tachyphylaxis, the participants were first exposed to the odor for 4 min on the day before the experiment. The time point at which the odor was no longer perceived was recorded and used in the design of the experimental protocol.

#### 3.3.2. Sociodemographic Data Form

The questionnaire collected a range of sociodemographic data, including participants’ age, duration and level of education, marital status, academic affiliation, and current employment status. Additionally, both personal medical history and relevant family health information were obtained through a structured questionnaire.

#### 3.3.3. Handedness Scale

The questionnaire asked participants to specify their hand preference for tasks typically done with one hand, such as writing, drawing, and throwing. It consisted of 13 items and had a maximum possible score of 39. Higher scores indicated a greater degree of left-handedness, with scores lower than 17 indicating right-handedness [52,53].

#### 3.3.4. Beck Anxiety Inventory

In the present study, the Beck Anxiety Inventory was used to assess anxiety symptoms.

This self-report instrument is designed to evaluate how frequently individuals experience symptoms related to anxiety. Comprising 21 items, each statement is rated on a four-point Likert scale ranging from 0 (not at all) to 3 (severely). The Turkish adaptation and validation of the scale were performed by [55]. Based on the cumulative score, anxiety severity is categorized as follows: scores below 10 suggests minimal or no anxiety, scores of 10–18 reflects mild anxiety, scores of 19–29 indicates moderate anxiety, and scores between 30 and 63 point to severe anxiety [54,55].

#### 3.3.5. Beck Depression Inventory

This scale is designed to assess the presence and severity of depressive symptoms in adults, as well as to monitor changes over time. The Turkish adaptation and psychometric validation were conducted by Hisli, and the cut-off score was established at 17. The instrument yields a total score ranging from 0 to 63. Based on the total scores, symptom severity is categorized as follows: 10–16 reflects mild emotional distress, 17–20 suggests subclinical depression, 21–30 corresponds to moderate depression, 31–40 indicates severe depression, and scores above 41 are indicative of very severe depression [56].

#### 3.3.6. Adult ADD/ADHD DSM-IV-Based Diagnostic Screening and Rating Scale

Originally developed by Turgay in 1995 in Canada, the Adult ADHD scale utilizes a five-point Likert-type format and is organized into three distinct sections. The first section includes 9 items assessing the DSM-IV attention deficit (AD) symptoms, whereas the second section includes 9 items assessing the DSM-IV hyperactivity symptoms. The third section consists of 30 items developed on the basis of clinical experience and observations. Overall, total scores below 20 indicate low levels of ADHD, scores between 20 and 59 indicate moderate levels of ADHD, and scores above 59 indicate high levels of ADHD symptoms [57,58].

### 3.4. Essential Oil Administration and Experimental Procedure

Lavender, rosemary, lemon, and peppermint essential oils used in this study were selected from pharmacopoeial-quality batches that had previously undergone comprehensive quality control analyses, including pharmacopoeial testing and GC–MS characterization. The botanical identity, production method, storage conditions, pharmacopoeial compliance, and principal composition of the essential oils are summarized in Table 7. Detailed compositional data were reported in our previous publication [29].

**Figure 5:**
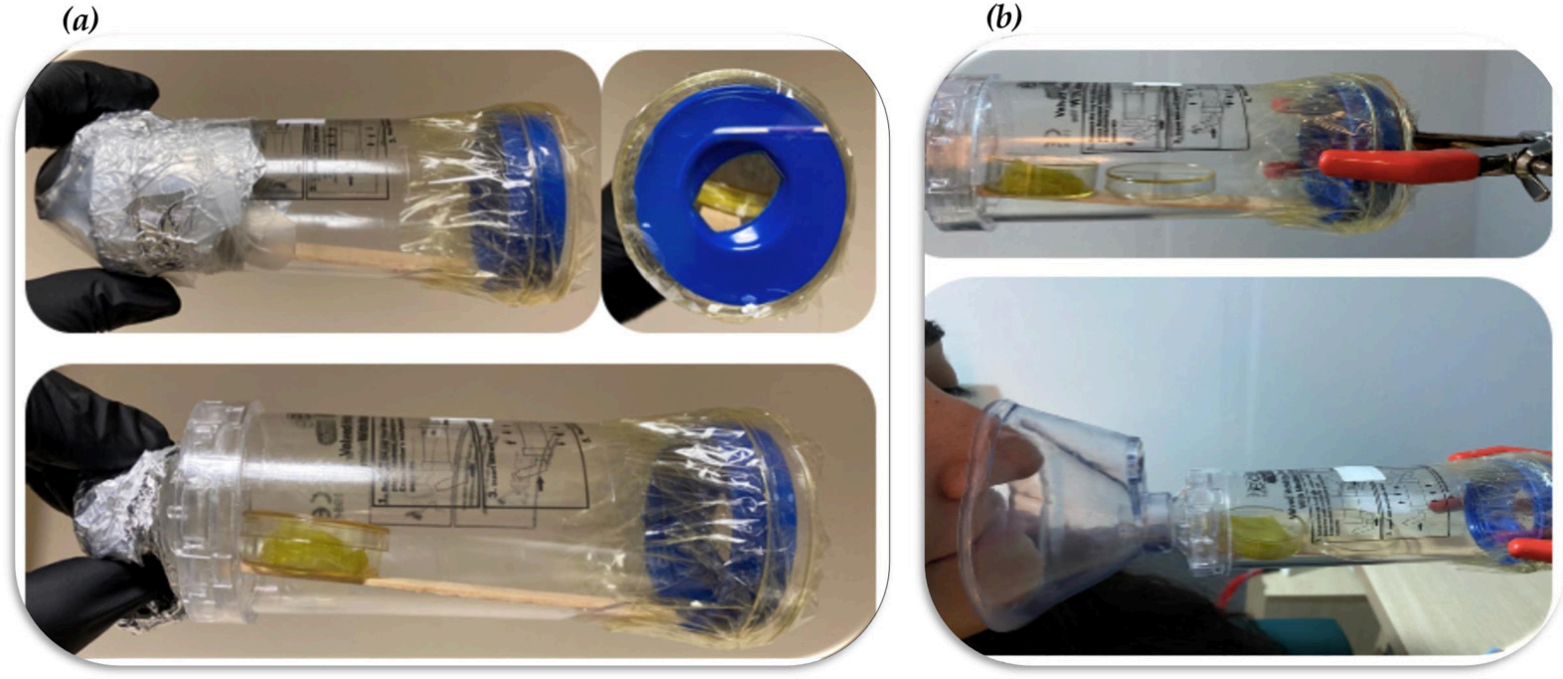
Inhalation chamber used in the EEG experiment: (**a**) Petri dish containing filter paper impregnated with the essential-oil preparation; (**b**) chamber positioned for inhalation exposure.

Four essential oil samples (lavender, rosemary, lemon, peppermint) and a mixture (rosemary:lemon:peppermint at a ratio of 1:1:1) were used as the test materials, while grape-seed oil served as the control. For each odor condition, 1 mL of essential oil (or the essential-oil mixture) was mixed with 1 mL of grape-seed oil (1:1, *v*/*v*), resulting in a final essential-oil concentration of 50% (*v*/*v*). The entire 2 mL preparation was applied to filter paper placed in a 35-mm Petri dish inside an AeroChamber spacer (Exxe Chamber, BS Başar Health Products Medical Industry and Trade Ltd. Co., Istanbul, Türkiye, internal volume: 216 mL). A new AeroChamber, filter paper, and Petri dish were used for each odor condition to prevent cross-contamination. EEG data were recorded with participants’ eyes closed, in the seated position, in a dimly lit, sound-attenuated room. The room temperature was kept at 25 ± 1 °C, and the humidity level was maintained at 10%, continuously monitored under both experimental and control conditions. Participants were instructed to breathe normally for 3 min with eyes closed, in a sitting position. The odor was administered using a specially designed AeroChamber positioned approximately 10 cm from the participant’s nose (Figure 5). Airflow through the AeroChamber was not measured or actively controlled. Likewise, the volatile concentration in the participant’s breathing zone and the actual inhaled dose were not quantified.

Following an initial 3-min resting-state EEG recording with participants’ eyes closed and without the inhalation chamber, participants completed five control–odor pairs, resulting in a total of 11 recording sessions (1 resting-state recording, 5 grape-seed oil control recordings, and 5 essential-oil recordings). Each essential-oil condition was preceded by a separate grape-seed oil control recording; therefore, control recordings were obtained independently for each odor condition and were not reused across different odor conditions. Within each control–odor pair, the sequence was fixed, with grape-seed oil administered before the corresponding essential oil. The order of the five essential-oil conditions (lavender, rosemary, lemon, peppermint, and the essential-oil mixture) was randomized across participants using the Research Randomizer program. Between successive control–odor pairs, participants rested for 3 min while the inhalation chamber was removed from the experimental room and the room was ventilated. This interval served as the washout period between successive odor conditions.

### 3.5. Electroencephalogram (EEG) Recording

EEG recordings were obtained using the BrainAmp DC 32-channel EEG-EP system (Brain Products GmbH, Gilching, Germany), employing 30 Ag-AgCl active electrodes (including Fz, F3, F4, F7, F8, FCz, etc.) arranged on an elastic cap according to the extended 10–20 international electrode placement system. The FCz electrode was used as the reference electrode during recording. During the analysis, M1 and M2 electrodes placed over the mastoid processes behind the ears were assigned as references. The ground electrode was positioned centrally on the forehead, corresponding to the region between the FP1 and FP2 electrode sites. Electrooculographic (EOG) activity was recorded using electrodes positioned at the outer canthi and the supraorbital area of the left eye. Both EEG and EOG signals underwent digital amplification and were sampled at a rate of 1000 Hz. Throughout data acquisition, electrode impedance levels were maintained below 10 kΩ.

#### EEG Data and Statistical Analysis

EEG recordings were processed using the BrainVision Analyzer 2.1 software (Brain Products, GmbH, Gilching, Germany). Spectral power was computed via Fast Fourier Transform (FFT) for the theta (4–8 Hz), alpha-1 (8–10 Hz), and alpha-2 (10–12 Hz) frequency bands. These frequency bands were selected for detailed evaluation because previous aromatherapy EEG studies have most consistently reported effects within these ranges. Although additional frequency bands were examined during exploratory analyses, no statistically significant alterations were observed; therefore, the present study focused on the bands showing the most relevant neurophysiological responses. Prior to analysis, the raw EEG signals were filtered using a 0.5–30 Hz band-pass filter. To eliminate ocular and movement-related artifacts, Independent Component Analysis (ICA) was employed [59]. The continuous data stream was segmented into 103 non-overlapping epochs, each lasting 2.048 s. FFT calculations utilized a Hanning window with a 10% taper and frequency resolution of 0.5 Hz.

The electrodes were grouped into the following regions: FronR (Right Frontal Region: F4, F8), FronL (Left Frontal Region: F3, F7), TempR (Right Temporal Region: T8, TP8), TempL (Left Temporal Region: T7, TP7). For every region, the mean of the power values of electrodes was calculated. The log10-transformed EEG power ratios [log10(target oil/control)] were used for statistical analysis. For each frequency band, a three-way repeated-measures analysis of variance (ANOVA) was conducted with oil condition (lavender, rosemary, lemon, peppermint, and mixed oil), cortical region (frontal and temporal), and hemisphere (left and right) as within-subject factors. Where appropriate, pairwise comparisons were performed using the Bonferroni adjustment to control for multiple comparisons. Given the normal distribution of the data, follow-up analyses comparing each experimental oil condition with its corresponding grape-seed oil control condition were conducted using paired-samples *t*-tests. Because EEG frequency bands such as theta, alpha, and beta are associated with partially distinct neurophysiological and cognitive processes, and because their absolute power values may differ substantially in scale and distribution [60], statistical analyses were conducted separately for each predefined frequency band. This approach allowed band-specific effects of each oil condition relative to the grape-seed oil control condition to be evaluated more clearly.

All statistical computations were carried out using IBM SPSS software, version 28 (IBM Corp., Armonk, NY, USA, 1.0.0.1447). The assumption of normality for EEG data was evaluated through the Shapiro–Wilk test, which confirmed that the dataset conformed to a normal distribution.

**Figure 6:**
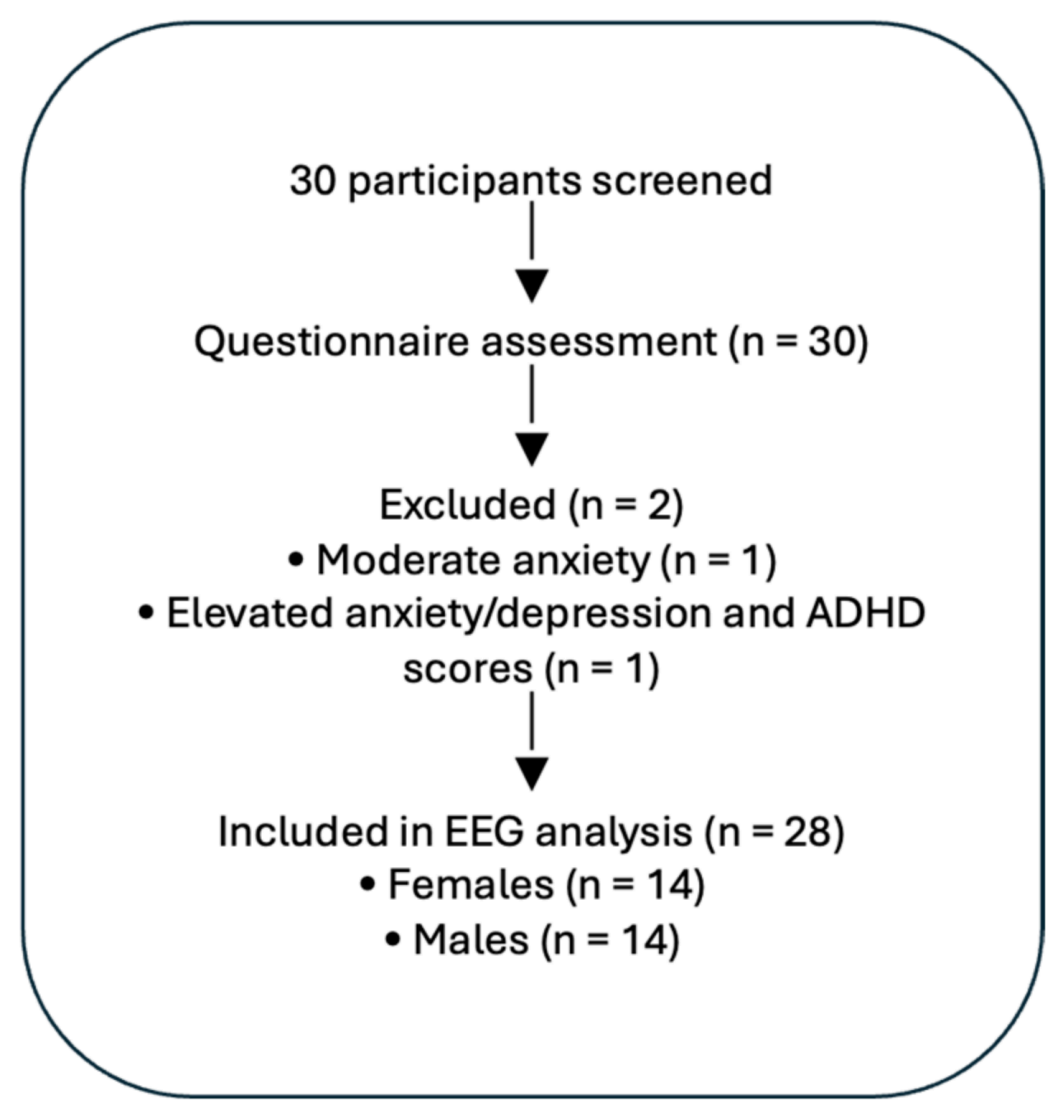
Flow diagram of participant selection for EEG analysis.

The exclusion criteria based on the Beck Anxiety Inventory, Beck Depression Inventory, and Adult ADHD DSM-IV-Based Diagnostic Screening and Rating Scale were defined before data analysis. The participant selection process is summarized in Figure 6. Questionnaire results were evaluated before EEG data analysis. Following an evaluation of individual questionnaire outcomes, two participants were excluded from the analysis. One displayed a moderate anxiety level, scoring 23 on the Beck Anxiety Inventory, while the other exhibited elevated scores of 24 on both the Beck Anxiety and Depression Inventories, along with a score of 46 on the Hyperactivity Scale.

The final analysis was therefore performed using data from the remaining 28 participants.

Subsequent analyses were carried out using data from the 28 participants who met all inclusion criteria. The final sample was balanced by sex comprising 14 female and 14 male individuals.

### 3.6. Preparation of Microemulsions

For the preparation of microemulsions, lavender essential oil, rosemary essential oil, lemon essential oil, peppermint essential oil, Span 80, ethanol, propylene glycol, and distilled water were used. Span 80 was employed as the surfactant in all formulations. Ethanol was used as the co-surfactant in the M1 formulation, whereas propylene glycol served as the co-surfactant in the M2 and M3 formulations. Stepwise titration of pre-equilibrated oil/surfactant/co-surfactant blends with water at a constant addition rate delineated the pseudo-ternary phase diagrams. Before aqueous titration commenced, the surfactant and co-surfactant fractions were determined on a weight-to-weight basis and were thoroughly homogenized with the oil phase. Dropwise addition of distilled water titrated the pre-equilibrated mixture. Stirring continued at 150 rpm and 25 ± 2 °C until turbidity emerged. The resulting compositional data were subsequently plotted on a pseudo-ternary phase diagram comprising the oil, surfactant/co-surfactant, and water fractions. Within this diagram, the ideal microemulsion region was delineated by the centroid of the plot.

#### Characterization of Microemulsions

The physicochemical attributes of microemulsions were comprehensively characterized. At 25 ± 2 °C, they determined pH values with a precision pH meter (Mettler Toledo, Columbus, OH, USA). Under identical thermal conditions, a refractometer (Krüss, Hamburg, Germany) provided refractive index measurements. Photon correlation spectroscopy (Nano ZS, Malvern Instruments, Worcestershire, UK) subsequently quantified droplet diameter, polydispersity index, and zeta potential. At 25 ± 2 °C, electrical conductivity for each microemulsion was quantified with a calibrated bench-top conductometer (Model DDS-307, Mettler-Toledo, Shanghai, China). Rheological characterization was conducted using a rotational viscometer (DV II+ Pro, Brookfield Engineering Laboratories, Middleborough, MA, USA) under the same thermal conditions, whereby determination of apparent viscosity provided detailed insight into the formulations’ flow behaviour [35]. The Dynamic Light Scattering was carried out to determine the droplet size and PDI using a Malvern Zetasizer Nano ZS at 25 ± 2 °C.

The stability studies of the M1, M2 and M3 formulations were conducted under three different storage conditions, 5 ± 3 °C (in a refrigerator), 25 ± 2 °C, and 30 ± 2 °C, for a period of 3 months.

### 3.7. Cell Culture and MTT Assay

Cell culture reagents were obtained from Gibco, UK. MTT salt was obtained from Sigma. HaCaT human keratinocyte cells were maintained in high-glucose Dulbecco’s Modified Eagle’s Medium (DMEM/High), enriched with 10% (*v*/*v*) heat-inactivated fetal bovine serum, L-glutamine, and a standard antibiotic–antimycotic mix (penicillin 100 U/mL, streptomycin 100 μg/mL, and amphotericin B 0.25 μg/mL). Cultures were incubated at 37 °C under humidified conditions with 5% CO_2_ and passaged every three days to sustain cell viability and growth [61,62,63].

Cell viability was assessed using the MTT assay following a standard protocol. Twenty-four hours prior to treatment, cells were plated onto flat-bottomed 96-well microplates at a seeding density of 1 × 10^5^ cells per well to allow for adherence and stabilization. This specific high seeding density was selected to ensure a robust and uniform monolayer of HaCaT keratinocytes, optimized for the high-throughput screening of dense microemulsion systems, while maintaining optical absorbance within the linear range of the microplate reader. The absolute viability of the untreated control groups remained stable throughout the 24-h incubation period, confirming that the cell density did not induce contact inhibition or premature senescence under the current experimental conditions. To assess the cytotoxicity of the tested formulations, cells were treated with increasing concentrations (0.1–2000 µg/mL) of each sample, prepared in DMEM/High medium. Cisplatin was employed as a positive cytotoxicity control under identical experimental conditions. After the treatment period, the culture medium was carefully removed, and 30 µL of MTT reagent (0.5 mg/mL in sterile D-PBS) was added to each well, followed by incubation for 4 h at 37 °C. Additionally, cell-free wells containing only the microemulsion formulations and MTT reagent were prepared and monitored under the same conditions as cell-free controls to exclude any direct chemical reduction of MTT or spectral absorbance interference by the samples. Then, 150 µL of isopropanol was introduced to solubilize the formazan precipitates, and absorbance values were recorded at 570 nm using a SpectraMax i3 microplate reader (Molecular Devices, LLC, San Jose, CA, USA) [64]. Each formulation was analyzed in triplicate across three independent experiments. The results were evaluated according to the following equation:

*y* = (*A_e_*/*A_c_*) × 100 (1)

*y: Viability%*

*A_e_: Absorbance of experimental group*

*A_c_: Absorbance of control group*

## 4. Conclusions

The existing literature indicates that olfactory stimuli may influence neurophysiological responses and subjective states. In the present study, exploratory pairwise analyses identified small, region-specific changes in theta, alpha-1, and alpha-2 power; however, none of these findings remained statistically significant after correction for multiple comparisons. Repeated-measures analyses likewise revealed no statistically significant main effects or interactions, although the main effect of test condition on alpha-1 power remained borderline after Greenhouse–Geisser correction (*p* = 0.053). No statistically significant sex-related differences were identified. These findings support the use of EEG as an objective exploratory tool for characterizing responses to essential oil inhalation but do not establish confirmatory oil-specific neurophysiological or functional effects. The developed microemulsion systems demonstrated suitable physicochemical stability and generally low cytotoxicity, providing a formulation framework that warrants further investigation. Because the developed formulations were not evaluated using the EEG protocol, comparable exposure and neurophysiological responses remain to be established in formulation-specific studies. Larger studies incorporating behavioral, psychometric, and clinical endpoints are needed to determine the functional relevance of the exploratory EEG patterns

## Figures and Tables

**Table 1:** Nominally significant paired-samples *t*-test results for theta power.

Oil Condition	Region/Hemisphere	Oil, M (SD)	Grape-Seed Control, M (SD)	Mean Difference	95% CI	t(27)	*p*
Lemon	Left frontal	0.433 (0.083)	0.414 (0.089)	0.0190	[0.0019, 0.0361]	2.28	0.031
Lemon	Left temporal	0.289 (0.108)	0.270 (0.124)	0.0192	[0.0005, 0.0379]	2.10	0.045

Note. Values are log10-transformed theta power. M = mean; SD = standard deviation; CI = confidence interval. Mean difference was calculated as essential oil minus its matched grape-seed control. Only nominally significant two-tailed comparisons (*p* < 0.05) are shown. After Bonferroni correction for 20 comparisons (adjusted *α* = 0.0025), none of the comparisons remained statistically significant.

**Table 2:** Nominally significant paired-samples *t*-test results for alpha-1 power.

Oil Condition	Region/Hemisphere	Oil, M (SD)	Grape-Seed Control, M (SD)	Mean Difference	95% CI	t(27)	*p*
Lavender	Left frontal	0.453 (0.190)	0.477 (0.174)	−0.0235	[−0.0424, −0.0046]	−2.55	0.017
Lavender	Right temporal	0.311 (0.265)	0.344 (0.219)	−0.0326	[−0.0596, −0.0056]	−2.48	0.020
Mixed oil	Right frontal	0.450 (0.183)	0.470 (0.175)	−0.0206	[−0.0339, −0.0073]	−3.18	0.004
Mixed oil	Left frontal	0.455 (0.189)	0.474 (0.177)	−0.0188	[−0.0375, −0.0002]	−2.07	0.048
Peppermint	Right frontal	0.444 (0.192)	0.466 (0.180)	−0.0214	[−0.0382, −0.0046]	−2.61	0.015

Note. Values are log10-transformed alpha-1 power. M = mean; SD = standard deviation; CI = confidence interval. Mean difference was calculated as essential oil minus its matched grape-seed control. Only nominally significant two-tailed comparisons (*p* < 0.05) are shown. After Bonferroni correction for 20 comparisons (adjusted α = 0.0025), none of the comparisons remained statistically significant.

**Table 3:** Nominally significant paired-samples *t*-test results for alpha-2 power.

Oil Condition	Region/Hemisphere	Oil, M (SD)	Grape-Seed Control, M (SD)	Mean Difference	95% CI	t(27)	*p*
Lavender	Right temporal	0.199 (0.193)	0.237 (0.152)	−0.0377	[−0.0709, −0.0045]	−2.33	0.027

Note. Values are log10-transformed alpha-2 power. M = mean; SD = standard deviation; CI = confidence interval. Mean difference was calculated as essential oil minus its matched grape-seed control. Only nominally significant two-tailed comparisons (*p* < 0.05) are shown. After Bonferroni correction for 20 comparisons (adjusted *α* = 0.0025), none of the comparisons remained statistically significant.

**Table 4:** Composition of the developed microemulsion-based formulations (g).

Formulation Code	Lavender Oil	Grape-Seed Oil	Rosemary:Lemon:Peppermint EO (1:1:1)	Span 80	Ethanol	Propylene Glycol	Water
M1	4.122	-	-	1.028	4.112	-	0.738
M2	4.553	0.911	-	3.299	-	1.100	0.137
M3	-	-	5.623	3.485	-	0.697	0.195

M1: Microemulsion-based Pillow Spray; M2: Microemulsion-based Topical Patch-1; M3: Microemulsion-based Topical Patch-2. EO: Essential Oil.

**Table 5:** Characterization results of M1 (Pillow Spray), M2 (Lavender Topical Patch) and M3 (Rosemary:Lemon:Peppermint Topical Patch) formulations.

Parameters	M1	M2	M3
pH	6.890 ± 0.007	6.756 ± 0.05	7.04 ± 0.0054
Viscosity (cP)	4.520 ± 0.042	40.100 ± 7.449	24.6 ± 0.05
Refractive index	1.4182 ± 0.0002	1.4644 ± 0.0008	1.46588 ± 0.0002
Electrical conductivity (μS)	0.0365 ± 0.00007	0.00965 ± 0.0102	0 ± 0
Droplet size (nm)	1.106 ± 0.015	9.047 ± 1.475	92.19 ± 11.053
PDI	0.209 ± 0.017	0.500 ± 0.040	0.347 ± 0.025
Zeta potential (mV)	−0.414 ± 0.515	−0.130 ± 0.0295	−0.057 ± 0.0021

All values are expressed as mean ± SD.

**Table 6:** Stability results of the microemulsion formulations (M1, M2 and M3) after 3 months of storage in stability chambers at 5 ± 3 °C (refrigerated), 25 ± 2 °C/60 ± 5% RH, and 30 ± 2 °C/65 ± 5% RH.

Storage Condition	Measured Parameter	M1 (Pillow Spray)	M2 (Lavender Topical Patch)	M3 (Rosemary:Lemon:Peppermint Topical Patch)
5 ± 3 °C (in refrigerator)	Appearance	Suitable	Suitable	Suitable
	pH	6.81 ± 0.002	6.7 ± 0.04	7 ± 0.005
	Viscosity	4.6 ± 0.001	39.4 ± 1.15	24.2 ± 0.03
	Electrical Conductivity	0.85 ± 0.00001	0.0009 ± 0.01	0 ± 0
	Refractive Index	1.4177 ± 0.0001	1.4635 ± 0.0003	1.4678 ± 0.0001
	Droplet Size	1.115 ± 0.008	9.047 ± 1.475	102.05 ± 10.003
	Polydispersity Index	0.239 ± 0.012	0.503 ± 0.034	0.398 ± 0.018
	Zeta Potential	−0.084 ± 0.005	−0.039 ± 0.0002	−0.027 ± 0.0001
25 ± 2 °C	Appearance	Suitable	Suitable	Suitable
	pH	6.7 ± 0.004	6.72 ± 0.03	6.9 ± 0.002
	Viscosity	4.5 ± 0.03	38.5 ± 1.86	24.5 ± 0.02
	Electrical Conductivity	0.92 ± 0.00001	0.0005 ± 0.01	0 ± 0
	Refractive Index	1.4180 ± 0.0002	1.4630 ± 0.0002	1.4655 ± 0.0003
	Droplet Size	1.107 ± 0.002	10.002 ± 1.303	98.12 ± 0.931
	Polydispersity Index	0.288 ± 0.022	0.510 ± 0.027	0.405 ± 0.035
	Zeta Potential	−0.397 ± 0.001	−0.100 ± 0.001	−0.171 ± 0.002
30 ± 2 °C	Appearance	Suitable	Suitable	Suitable
	pH	6.3 ± 0.001	6.5 ± 0.01	6.7 ± 0.003
	Viscosity	4.9 ± 0.002	46.2 ± 2.34	27 ± 0.04
	Electrical Conductivity	10 ± 0.00001	0.0004 ± 0.01	0 ± 0
	Refractive Index	1.4192 ± 0.0001	1.4625 ± 0.0001	1.4692 ± 0.0001
	Droplet Size	1.103 ± 0.012	10.027 ± 1.345	99.21 ± 10.004
	Polydispersity Index	0.311 ± 0.024	0.513 ± 0.058	0.442 ± 0.046
	Zeta Potential	−0.348 ± 0.001	−0.403 ± 0.004	−0.043 ± 0.0001

**Table 7:** Characteristics of the essential oils used in the study.

Essential Oil	Accepted Botanical Name	Plant Part	Extraction Method	Supplier (Lot No.)	Principal Constituents (%)
Lavender	*Lavandula angustifolia* Mill.	Flowering tops	Steam distillation	Local producer, Isparta, Türkiye (Lot No.: not available)	Linalyl acetate (33.5%), linalool (22.3%), (Z)-β-ocimene (11.6%), β-caryophyllene (5.1%), terpinen-4-ol (4.6%), lavandulyl acetate (4.5%)
Rosemary	*Rosmarinus officinalis* L.	Flowering aerial parts	Steam distillation	Manolya, Isparta, Türkiye (Lot No.: not available)	1,8-Cineole (48.0%), camphor (13.5%), α-pinene (10.3%), β-pinene (7.5%), camphene (4.2%), limonene (4.1%)
Lemon	*Citrus limon* (L.) Osbeck	Fresh fruit peel	Cold expression	Manolya, Isparta, Türkiye (Lot No.: not available)	Limonene (70.7%), β-pinene (10.0%), γ-terpinene (7.4%)
Peppermint	*Mentha* × *piperita* L.	Fresh aerial parts	Steam distillation	PharmaTerm, İstanbul, Türkiye (Lot No.: 485340875320)	Menthol (43.0%), menthone (30.0%), 1,8-cineole (5.9%), menthyl acetate (5.2%), isomenthone (4.5%)

Essential oils were stored in tightly closed amber glass bottles at 4 °C and protected from light until use. All essential oils complied with the relevant monographs of the Turkish Pharmacopoeia (2017). The same GC–MS-characterized essential oil samples were used throughout both the EEG and microemulsion experiments.

## Data Availability

The data presented in this study are available from the corresponding author upon reasonable request.

## Abbreviations

**ADD**—Attention Deficit Disorder; **ADHD**—Attention Deficit Hyperactivity Disorder; **ANOVA**—Analysis of Variance; **cP**—Centipoise (viscosity unit); **DMEM**—Dulbecco’s Modified Eagle’s Medium; **D-PBS**—Dulbecco’s Phosphate-Buffered Saline; **EEG**—Electroencephalography/Electroencephalogram; **EOG**—Electrooculography; **FFT**—Fast Fourier Transform; **HaCaT**—Human Adult Low Calcium High Temperature keratinocyte cell line; **Hz**—Hertz; **ICA**—Independent Component Analysis; **IC_50_**—Half-maximal inhibitory concentration; **kg/m^2^**—Kilogram per square meter (body mass index unit); **M1, M2, and M3**—Microemulsion formulations developed in the study; **MTT**—3-(4,5-Dimethylthiazol-2-yl)-2,5-diphenyltetrazolium bromide (assay for cytotoxicity); **nm**—Nanometer; **PDI**—Polydispersity Index; **rpm**—Revolutions per minute; **SD**—Standard Deviation; **SPSS**—Statistical Package for the Social Sciences; ***v*/*v***—Volume/olume ratio.
